# Assessing Deception in Questionnaire Surveys With Eye-Tracking

**DOI:** 10.3389/fpsyg.2021.774961

**Published:** 2021-11-22

**Authors:** Xinyue Fang, Yiteng Sun, Xinyi Zheng, Xinrong Wang, Xuemei Deng, Mei Wang

**Affiliations:** ^1^School of Mechanical Engineering, Sichuan University, Chengdu, China; ^2^School of Design, South China University of Technology, Guangzhou, China

**Keywords:** lie detection, eye behavior, questionnaire surveys, deception, eye-tracking

## Abstract

Deceit often occurs in questionnaire surveys, which leads to the misreporting of data and poor reliability. The purpose of this study is to explore whether eye-tracking could contribute to the detection of deception in questionnaire surveys, and whether the eye behaviors that appeared in instructed lying still exist in spontaneous lying. Two studies were conducted to explore eye movement behaviors in instructed and spontaneous lying conditions. The results showed that pupil size and fixation behaviors are both reliable indicators to detect lies in questionnaire surveys. Blink and saccade behaviors do not seem to predict deception. Deception resulted in increased pupil size, fixation count and duration. Meanwhile, respondents focused on different areas of the questionnaire when lying versus telling the truth. Furthermore, in the actual deception situation, the linear support vector machine (SVM) deception classifier achieved an accuracy of 74.09%. In sum, this study indicates the eye-tracking signatures of lying are not restricted to instructed deception, demonstrates the potential of using eye-tracking to detect deception in questionnaire surveys, and contributes to the questionnaire surveys of sensitive issues.

## Introduction

Questionnaire is one of the most widely used tools for data collection due to its wide range of applications, flexibility, speed, and convenience (Taherdoost, 2016). However, there is subjectivity and freedom when filling out questionnaires. Thus, answers to sensitive questions are often distorted (such as evaluations of self or others, substance use, sexual activities, political opinions, unsocial attitudes) (Holtgraves, 2004; Krumpal, 2013). The respondents will go through five stages when answering a self-report: (1) Explain the question. (2) Retrieve information. (3) Generate an opinion. (4) Format a response. (5) Edit the response (Sudman et al., 1997). The effect of social desirability usually operates at the final editing stage (Tourangeau and Rasinski, 1988; Sudman et al., 1997; Holtgraves, 2004). Respondents weigh the benefits and risks of telling the truth. When the risks are higher than the benefits, the respondent will choose to lie (Tourangeau et al., 2000; Walzenbach, 2019). Respondents can exaggerate, minimize, omit, and present themselves in a socially desirable light (Bond and DePaulo, 2006; Rauhut and Krumpal, 2008; Preisendörfer and Wolter, 2014; Walzenbach, 2019). Accordingly, lying in surveys can lead to misreported data and reduce the reliability of the findings. Especially, research on sensitive questions is the most likely area of survey misreporting (Lensvelt-Mulders, 2008; Preisendörfer and Wolter, 2014). Fortunately, the design of questionnaires (such as expressions) can change the sensitivity of questions, which will have a massive impact on people’s responses when filling out the questionnaire (Walzenbach, 2019). Thus, it is essential to identify and modify the questions in the questionnaire that tend to cause lying. Lie detection in questionnaire surveys helps to improve the design of questionnaires before they are published, and to avoid using unreliable results.

Lie detection has been a source of fascination. Even though detecting lies is necessary, the accuracy of human detection of lies is around the chance level, with an average of 54% (Bond and DePaulo, 2006). Deception is usually thought to be correlated with cognitive load. There are three main theoretical perspectives on the relationship between deception and cognitive load. The first theoretical perspective is that lying will experience more complex cognitive processes and bear a higher cognitive load than honesty (Zuckerman et al., 1981; Vrij et al., 2001, 2017; Roma et al., 2018). People will modify the answers that meet social desirability at the response editing stage, and there is more hesitation (Holden et al., 2001; DePaulo et al., 2003). The second theoretical perspective is the opposite (Holden et al., 1985; Leary and Kowalski, 1990). When lying, the respondents do not need to recall accurate information, they directly respond according to social desirability and do not move through the retrieve information stage. The third theoretical perspective suggests that response time depends on the lying schema and the social desirability of the test item (Brunetti et al., 1998; Holden et al., 2001). A previous study conducted a meta-analysis of 26 cognitive lie detection studies with a weighted mean of 74% overall accuracy (Vrij et al., 2017). Whereas, to date, most studies investigating lie detection have focused on face to face communication, such as criminal justice scenarios (Porter and ten Brinke, 2010; Synnott et al., 2015; Vrij and Fisher, 2016) and conversation scenarios (Vrij, 2018; Nahari and Nisin, 2019). The literature investigating the questionnaire surveys without verbal cues is not as rich. Moreover, in the field of lie detection in questionnaire surveys, most studies have only focused on lie detection on personality tests (van Hooft and Born, 2012; Mazza et al., 2020). However, questionnaires cover a wide range of areas, not just limited to personality tests. But up to now, far too little attention has been paid to lie detection in broader questionnaire areas.

Extensive studies about lie detection were limited to simulated scenarios, where participants were instructed to lie. Nevertheless, when instructed to lie, participants’ motivations are low, and they probably do not have any concern with the accuracy and need not fear their behaviors are detected (von Hippel and Trivers, 2011; van Hooft and Born, 2012). For this, several authors have proposed that deception detection studies should be conducted in a more ecological way (Wright et al., 2013; Levine, 2018). As Ganis et al. (2003) and Yin et al. (2016) discussed, there are different patterns of activation while expressing rehearsed or spontaneous lies in fMRI. Furthermore, Delgado-Herrera et al. (2021) performed a meta-analysis of fMRI deception tasks through a review from 2001 to 2019, and the results showed that the tasks with low ecological validity and high ecological validity lead to different areas of brain activation, perhaps because the tasks with high ecological validity are more realistic, and engage a broader network of brain mechanisms. In contrast, the Concealed Information Test results of Geven et al. (2018) showed no significant differences in skin conductance, heart rate, and respiration between spontaneous deception and instructed deception. Ask et al. (2020) found that instructed lies have little effect on human lie-detection performance. Whether the findings of the deception detection for instructed lies can be applied to reality remains controversial. There may be discrepancies between the mental processes of instructed lying and spontaneous lying in real life.

Eye-tracking is often considered an ideal measure for lie detection, as eye behaviors are automatic physiological responses that cannot be consciously controlled (Chen et al., 2013; Gonzales, 2018; Berkovsky et al., 2019). Eye-tracking is an appealing sensor for deception detection in questionnaire surveys, as it does not require direct physical contact (which may disturb the respondents), is easy to use, collects diversified information and can be used in automated screening systems (Cook et al., 2012; Proudfoot et al., 2015; Zi-Han and Xingshan, 2015; Ye et al., 2020). Previous studies showed that eye behaviors reflect people’s cognitive load (Zagermann et al., 2016), emotions (Zheng et al., 2014; Perkhofer and Lehner, 2019; Lim et al., 2020), attention (Lee and Ahn, 2012; Tsai et al., 2012), information processing (Bruneau et al., 2002). High cognitive load usually causes pupil dilation, decreased blink rate, increased saccade velocity and fixation duration (Wang et al., 2014; Zagermann et al., 2016; Einhäuser, 2017; Behroozi et al., 2018; van der Wel and van Steenbergen, 2018; Keskin et al., 2019, 2020). Arousal changes can affect blinks, saccades and fixations (Maffei and Angrilli, 2018), vigilance and fatigue can be detected in saccades, and information process can be predicted in saccades and fixations (Bruneau et al., 2002; Maffei and Angrilli, 2018). Fixation location can indicate the area of current focus (Rudmann et al., 2003). These all help to analyze the mental processes of deception. Furthermore, many studies have applied eye-tracking to detect deception with promising results. Deception changes people’s fixation patterns (Twyman et al., 2014). When lying, the pupil diameter becomes larger due to cognitive load, memory retrieval, vigilance, anxiety, etc. (Twyman et al., 2013; Proudfoot et al., 2016). Vrij et al. (2015) concluded that memory retrieval is greater when lying, so the saccade velocity is higher. George et al. (2017) found that the blink duration and blink count are higher when lying. Webb et al. (2009) suggested that people experience greater arousal when lying, resulting in greater pupil dilation and blink frequency. Borza et al. (2018) analyzed the eye movements to detect deception and obtained an accuracy of 99.3% on the dataset. van Hooft and Born (2012) found that on the personality test, more fixations occurred on the extreme response options when lying, while more fixations occurred on the middle response options when lying honest. They achieved 82.9% lie detection accuracy with eye-tracking. Consequently, eye behaviors attract more attention as psychological and physiological indicators of lying (Bessonova and Oboznov, 2018).

In summary, few studies investigated lie detection in questionnaire surveys, and the mental processes of spontaneous lying may not be identical to that of being instructed to lie. Therefore, this study simulated the scene of evaluating teachers to explore whether the subtle reaction of lying could be identified by eye-tracking in the questionnaire research scenario, and examined whether the changes in eye behaviors during instructed lying can be generalizable to spontaneous lying. In Study 1, the relationship between eye-tracking indicators and deception was initially explored, following the study of van Hooft and Born (2012), the participants were instructed to lie or be honest. We hypothesized that there would be significant differences in eye behaviors between lying and honesty condition in Study 1, which is consistent with the study of van Hooft and Born (2012). However, spontaneous lying in actual situations may cause more diverse mental processes. Consequently, we designed Study 2 to test whether the relationship between eye-tracking indicators and deception is still valid in the actual situation. In Study 2, this study created the motivation for participants to lie, and encouraged participants to lie spontaneously and genuinely. Study 2 investigated the eye behaviors when lying in the actual situation and compared them to the findings of Study 1 to examine if the eye behaviors that appeared in instructed lying still exist in spontaneous lying, and thus identify reliable eye movement indicators for detecting lies. In Study 2, our main hypothesis is that eye-tracking can effectively help to detect deception in questionnaire surveys in realistic situations. The present study has explored whether the eye behaviors in instructed lying can be generalized to reality, found reliable variables for lie detection in the actual situation, and could contribute to understanding the relationship between deception and eye behaviors. Moreover, this study confirmed the potential of eye-tracking in non-verbal lie detection, offered implications for detecting deception in questionnaire surveys.

## Study 1: Instructed Lie

### Materials and Methods

#### Scenario

A scenario was set to ask participants to evaluate their teachers. Chinese students are generally respectful of their teachers and desire to please their parents, teachers, and other people in positions of power (Bear et al., 2014). Chinese cultural expectations of the teacher–student relationship are “well-defined, rigidly hierarchical and authoritarian” (Ho and Ho, 2008). As the old Chinese idiom says, “once my teacher, forever my parents.” Students should respect their teachers as they respect their parents, including showing obedience (Hui et al., 2011). Respect for teachers is a revered virtue in China. Chinese students have high respect for those who provide knowledge and avoid challenging authority (Wei et al., 2015). Meanwhile, when evaluating leaders, students often worry that their teachers can be able to view their evaluations and thus judge them negatively. Hence, most students will choose to make no bad comments in real-name conditions to prevent adverse effects.

Participants were asked to recall a teacher they disliked. Then they were instructed to fill out the questionnaire according to the actual situation and imagine that the evaluation was in real-name condition.

#### Materials

**FIGURE 1:**
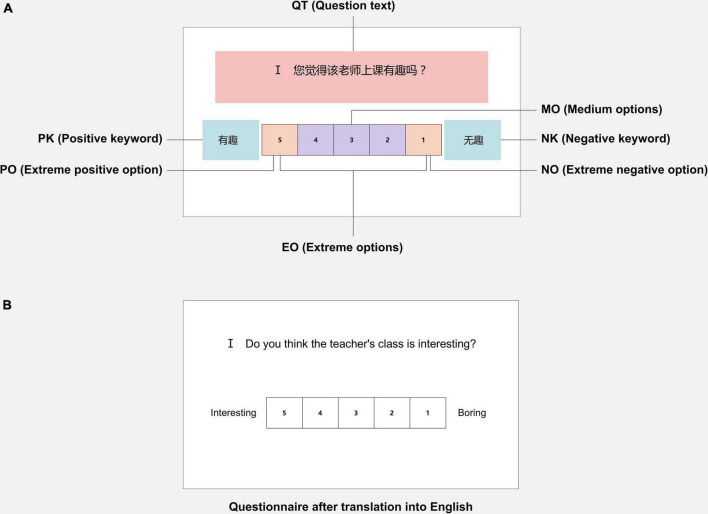
Example of stimuli. **(A)** Example of the questionnaire with marked AOIs. **(B)** Example of the questionnaire after translation into English. AOIs, areas of interest.

A questionnaire was designed for teacher evaluation. The questionnaire consists of 10 questions, including the evaluation of teaching level and attitude toward the teachers. A five-point scale was used in the study, with negative and positive keywords on either side of the options. Furthermore, this study defined several areas of interest (AOIs). The question text (QT), the extreme negative option (NO), the negative keyword (NK), the extreme positive option (PO), the positive keyword (PK), the extreme options (EO), and the medium options (MO) were defined as boxes of interest. The questionnaire and marked AOIs are shown in Figure 1.

#### Apparatus

An SMI iView X^TM^ RED desktop system with a high spatial resolution (0.005°) and a sampling rate of 500 Hz (1-ms temporal resolution) was used to record the participants’ eye behaviors. The system includes an iView PC. The operator controls the experiment, a 22-in. display screen (pixel resolution 1680 × 1050) to show the experimental stimuli to the participants. And an eye-tracking module was installed under the display screen to track the real-time eye behaviors of the participants.

#### Participants

Thirty one participants, including 18 males and 13 females, were recruited from Sichuan University, aged 20–26 (*M* = 22.68). All participants were healthy, had normal or corrected-to-normal visions, and had no reported history of neurological or psychiatric disorders. All of them received a small honorarium for their participation.

#### Procedure

Firstly, participants were asked to recall a teacher whom they disliked and describe him/her simply. Then, participants were told that they would evaluate the teacher through questionnaires, and their eye behaviors were recorded. Afterward, participants were provided with instructions that directed them to respond honestly or to imagine responding under the condition of real-name evaluation. Each participant was required to answer the questionnaire in the above two situations. The instructions were adapted from previous studies (McFarland and Ryan, 2000; van Hooft and Born, 2012). To eliminate the influence of order, the order of lying and honesty is random. An irrelevant questionnaire would be interspersed between the two responses to eliminate learning effects.

The instruction for encouraging participants to respond honestly is as follows:

*You will be presented with ten questions with five response options. Please answer the questions as honestly as possible. Your answers remain confidential and will be used for research purposes only. For this study, we are interested in your honest answers, so please answer the following questions as accurately and honestly as possible.*

The instruction for directing participants to imagine evaluation as a real-name situation to respond is as follows:

*You will be presented with ten questions with five response options. Please imagine that the teacher you are evaluating can see your answers in real-name. For this study, we are not interested in your honest answers. Instead, for each question, please select the answer you think is more beneficial to you.*

**FIGURE 2:**
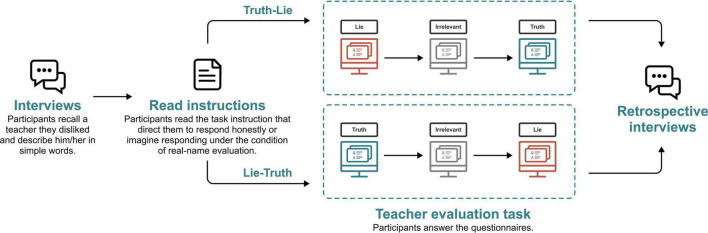
The experiment procedure of Study 1.

After understanding the requirements, Participants sat about 60 cm from the screen. After 2–4 times of eye-tracking calibration, the experimental material was displayed on the screen. The participants were required to respond to complete the evaluation questionnaire. By comparing the differences in the participants’ ratings, this study selected the questions that were rated differently. Afterward, we confirmed with participants whether the differences of rating in each question were caused by lying in the imagined real-name condition. The procedure of the experiment is shown in Figure 2.

#### Data Analysis

Eye movements were recorded and processed by Experiment Center 3.5 and BeGaze 3.5 on iView PC. Data analyses were performed using SPSS 26.0.

Each participant answered the questionnaire both in the condition of honesty and the condition of imagining the evaluation as a real-name situation. The questionnaire consisted of 10 questions, so each participant answered 10 pairs of questions, generating 10 paired data set. Participants’ ratings when instructed to be honest were considered to be true evaluations in this study. Most participants did not lie on every single question, so this study compared the two responses of questionnaires for differences in ratings. We removed the eye-tracking data for questions that did not rate higher in instructed lying condition than in honest condition, as well as the data of questions participants indicated that no lies existed. Meanwhile, we excluded eye-tracking data showing loss of eye movements and the extreme values that the boxplot indicated.

The normality of data was investigated using the Kolmogorov–Smirnov test. In the case of normal distributed data paired-sample *t*-test to test for the differences was performed. For the data out from normal, this study attempted a transformation, and conducted a paired-sample *t*-test to analyze variables that could be transformed to normal distributions. The transformation is according to the methods in the previous study (Coolican, 2014). For the variables that could not be transformed to normal distributions, Wilcoxon signed ranks test was performed. The significance level is 0.05. According to the previous studies (Fritz et al., 2011; Coolican, 2014; Tomczak and Tomczak, 2014), Cohen’s *d* is used to indicate the effect size of paired-sample *t*-test, and *r* is frequently used to indicate the effect size of Wilcoxon signed ranks test. In Study 1, paired-samples *t*-test was conducted for ratings and pupil size, Wilcoxon signed ranks test was conducted for blinks, saccades, and fixations.

### Results

#### Ratings

The results showed that 87.1% of the participants (27 of 31) chose to lie when imagining responding in the condition of real-name evaluation. In the real-name condition, the evaluation received higher ratings (*M* = 3.590, *SD* = 1.197) than in the honest condition (*M* = 2.255, *SD* = 1.211). A paired-samples *t*-test was conducted, the results revealed a significant difference between the ratings for the real-name condition and the honest condition (*t* = 15.861, Cohen’s *d* = 0.901, *p* < 0.001). 73.55% of all questions (288 of 310) were rated higher in the real-name condition, and were confirmed that lies existed by participants. Most participants responded slightly to completely different in the two conditions.

#### Pupil Size

As shown in Table 1, the results showed no significant differences in pupil size between lying and being honest (*p* = 0.722). However, when analyzing the participants’ pupil size in each area of interest, the results showed that the pupil size of who was lying was significantly larger than that of the participant who was honest in the MO area (Cohen’s *d* = 0.310, *p* = 0.001).

**TABLE 1:** The analysis of pupil size in Study 1.

Variable	Instructed lie		Truth		*t*	Effect size Cohen’s *d*	*P*-value (2-tailed)
	*M*	*SD*	*M*	*SD*			
Pupil diameter (mm)	4.243	0.456	4.239	0.409	0.356	0.026	0.722
Pupil diameter in the MO area (mm)	4.274	0.428	4.204	0.395	3.408	0.310	0.001**

Significance codes: **p < 0.01.

#### Blinks

This study found increasing blink count (*r* = −0.263, *p* < 0.001), blink frequency (*r* = −0.232, *p* = 0.001) and total blink duration (*r* = −0.006, *p* = 0.006) in lying, but no changes in average blink duration (*p* > 0.05), as shown in Table 2.

**TABLE 2:** The analysis of blink behaviors, saccade behaviors and fixation behaviors in Study 1.

Variable	Spontaneous lie		Truth		*Z*	Effect size *r*	*P*-value (2-tailed)
	*M*	*SD*	*M*	*SD*			
Blink count	2.258	2.829	1.535	1.717	−3.839	−0.263	< 0.001***
Blink frequency (count/s)	0.552	0.709	0.424	0.520	−3.364	−0.232	0.001**
Blink duration average (ms)	147.297	106.918	152.751	167.271	−0.085	−0.006	0.933
Blink duration total (ms)	425.851	610.696	314.900	396.193	−2.772	−0.190	0.006**
Saccade count	26.047	13.945	23.324	12.076	−2.720	−0.186	0.007**
Saccade frequency (count/s)	6.359	2.727	6.528	2.996	−1.408	−0.097	0.159
Saccade duration average (ms)	47.074	6.910	48.894	8.337	−2.578	−0.177	0.010*
Saccade duration total (ms)	1223.823	672.553	1136.381	627.435	−1.737	−0.130	0.082
Saccade velocity (°/s)	92.546	35.543	91.922	30.948	−2.578	−0.178	0.656
Saccade amplitude (°)	5.036	2.255	5.243	2.323	−1.569	−0.108	0.117
Fixation count	12.624	7.458	11.338	7.123	−2.506	−0.172	0.012*
Fixation frequency (count/s)	2.971	1.193	2.994	1.412	−1.000	−0.069	0.318
Fixation duration average (ms)	149.953	49.734	142.683	50.028	−2.015	−0.145	0.044*
Fixation duration total (ms)	2006.528	1231.404	1807.049	1143.751	−2.863	−0.206	0.004**

Significance codes: ***p < 0.001, **p < 0.01, *p < 0.05.

#### Saccades

There were no significant differences in frequency, total duration, velocity, and amplitude of saccade (*p* > 0.05) between deceptive and truthful responses, while there were significant differences in saccade count (*r* = −0.186, *p* = 0.007) and average saccade duration (*r* = −0.177, *p* = 0.010). Deception caused increased saccade count and decreased average saccade duration (see Table 2).

#### Fixations

As presented in Table 2, there was no significant difference in fixation frequency (*r* = −0.069, *p* > 0.05) between deceptive and truthful respond; by contrast, there were significant differences in fixation count (*r* = −0.172, *p* = 0.012), average fixation duration (*r* = −0.145, *p* = 0.044) and total fixation duration (*r* = −0.206, *p* = 0.004). Lies resulted in higher fixation counts, total fixation duration and average fixation duration.

This study analyzed the fixation behaviors in AOIs (see Table 3). In the NO and NK areas of lying participants, the fixation count (*r*_*NO*_ = −0.490, *r*_*NK*_ = −0.341, *p* < 0.001) and the percentage fixation time (*r*_*NO*_ = −0.521, *r*_*NK*_ = −0.400, *p* < 0.001) were lower than that of the honest one. In the PO and PK areas, the fixation count (*r*_*PO*_ = 0.522, *r*_*PK*_ = 0.326, *p* < 0.001) and the percentage fixation time (*r*_*PO*_ = 0.516, *r*_*PK*_ = 0.366, *p* < 0.001) were significantly higher in the lying condition. There were no significant differences in the QT, MO, and EO areas (*p* > 0.05).

**TABLE 3:** The analysis of fixation behaviors in the AOIs in Study 1.

AOI	Variable	Spontaneous lie		Truth		*Z*	Effect size *r*	*P*-value (2-tailed)
		*M*	*SD*	*M*	*SD*			
QT	Fixation count	6.799	4.349	6.995	4.553	−0.532	−0.038	0.595
	The percentage fixation time (%)	26.356	17.416	27.477	17.007	−0.895	−0.064	0.371
NO	Fixation count	0.144	0.455	0.814	1.123	−6.820	−0.490	<0.001***
	The percentage fixation time (%)	0.409	1.285	3.313	4.936	−7.259	−0.521	<0.001***
NK	Fixation count	0.362	0.731	0.701	0.829	−4.744	−0.341	<0.001***
	The percentage fixation time (%)	1.180	2.553	2.838	3.769	−5.568	−0.400	<0.001***
PO	Fixation count	1.026	1.491	0.460	0.216	−7.273	−0.522	<0.001***
	The percentage fixation time (%)	4.883	8.698	0.842	2.299	−7.187	−0.516	<0.001***
PK	Fixation count	0.881	1.239	0.464	0.789	−4.546	−0.326	<0.001***
	The percentage fixation time (%)	3.170	4.256	1.492	2.724	−5.094	−0.366	<0.001***
EO	Fixation count	1.170	1.481	1.057	1.188	−0.619	−0.044	0.536
	The percentage fixation time (%)	5.350	8.724	4.301	5.651	−0.472	−0.034	0.637
MO	Fixation count	2.959	2.639	2.696	2.556	−1.326	−0.095	0.185
	The percentage fixation time (%)	11.436	11.479	11.590	11.537	−0.039	−0.003	0.969

*AOI, area of interest; QT, the question text; NO, the extreme negative option; NK, the negative keyword; PO, the extreme positive keyword, PK, the positive keyword; EO, the extreme options; MO, the medium options.*

Significance codes: ***p < 0.001.

### Discussion

Under the instructions, the eye behaviors of participants between lying and being honest showed differences.

The results of Study 1 demonstrated that the participants who were instructed to lie had larger pupil size, higher count, frequency and duration of blink, higher count, velocity, and amplitude of saccade, as well as higher count and duration of fixation. The results of pupil size, saccades and fixations were consistent with the opinion that cognitive load is higher when lying. But the previous study suggested that the higher the cognitive load, the lower the blink rate (Zagermann et al., 2016). The present study observed that blink behaviors increased when lying, which means the cognitive is lower. However, the results of blink behaviors agreed with the findings that deception would result in greater blink count, blink frequency, and blink duration (Webb et al., 2009; George et al., 2017).

In the analysis of AOIs, when the participants were instructed to lie, the fixation count and the percentage fixation time were lower in the NO and NK areas, while higher in the PO and PK areas, compared to when they were honest. In the lying condition, the participants focused more on the positive items, while when being honest, they focused more on the negative items. It is in line with the answers of the questionnaire and mental processes. Nevertheless, there were no significant differences in fixation in the QT, MO, and EO areas.

## Study 2: Spontaneous Lie

In Study 1, this research found some eye-tracking indicators that differ when lying and being honest. However, in Study 1, participants were instructed to lie without real motivation. They did not need to worry about the accuracy of answers, the consequences of being discovered by the teacher, etc. Therefore, the mental processes of instructed lying may differ from the actual situation. In the actual situation, the pressure of evaluating teachers they dislike may lead to more complex mental processes and heavier cognitive loads.

Consequently, to explore whether the eye behaviors can help detect deception in the condition of spontaneous lying as in the condition of instructed lying, we implemented a new design for Study 2. This study simulated a more realistic scenario to identify whether the relationship between eye-tracking indicators and deception is still valid in the actual situation.

### Materials and Methods

#### Scenario

In the actual situation, people require motivation to lie. They choose to lie when the risks of telling the truth are higher than benefits (Tourangeau et al., 2000; Walzenbach, 2019). Participants were given scenarios in which telling the truth was risky to motivate them to lie. We continued to choose scenarios of evaluating teachers like Study 1. Participants were aware that the eye behaviors were recorded, but to motivate spontaneous lying, they were not aware of the purpose of the study. The participants were informed that this study was mainly assisting the school in gathering students’ evaluations of teachers, and also happened to conduct an eye-tracking study of the questionnaire reading processes. This study created realistic scenarios of evaluating teachers to observe participants’ performance of deception in the actual situation. We conducted interviews to ask participants to describe the teacher they disliked before the evaluations, and asked them for their names and student numbers to increase authenticity. After the experiment was completed, we explained the real purpose of the study to participants, and confirmed with participants whether they believed the scenario of real-name evaluation of teachers was real.

#### Materials

The content of the questionnaire used in Study 2 is the same as in Study 1. To increase the authenticity of the scenario, we added the school emblem to the questionnaire.

#### Apparatus

Same apparatus as Study 1.

#### Participants

35 participants were recruited from Sichuan University, aged 20–24(*M* = 21.77), 18 males and 17 females. All participants were healthy, had a normal or corrected-to-normal vision, and had no reported history of neurological or psychiatric disorders. All of them received a small honorarium for their participation.

#### Procedure

To eliminate the effects of order, the participants were randomly divided into two groups: the truth-lie group and the lie-truth group.

Truth-lie group:

(1) To explore the actual mental processes when evaluating the teachers, we *described the purpose of the study as exploring the relationship between eye behaviors and questionnaires*.
(2) An interview was conducted to ask each participant to describe a teacher they disliked the most during college life. We emphasized that the interview was anonymous and not recorded.
(3) We asked participants to fill out the questionnaire in front of the eye tracker. We *asked participants to fill in the questionnaires according to the interview content and to answer honestly.* The eye tracker recorded all eye behaviors during the questionnaire filling process.
(4) After completing the questionnaire, we explained: *In addition to the scientific study, our main purpose was to assist the school to investigate students’ satisfaction with the teachers. The questionnaire was real-name and would be recorded. Moreover, the teacher they evaluated could see the responses and respondents.* We asked participants for their names and student numbers to increase authenticity. *Participants were asked if they wanted to fill out the questionnaire again and invalidate the first one.* If participants agreed, they would complete the questionnaire a second time. All eye behaviors were recorded using an eye tracker. An irrelevant questionnaire would be interspersed between the two responses to eliminate learning effects.
(5) When participants completed the questionnaire or refused to fill out the questionnaire again, we explained that the scenario of evaluating teachers was simulated, and told them the study’s actual purpose. The responses of the questionnaire would be completely confidential and anonymous.
(6) We confirmed with participants whether they believed the scenario of real-name evaluation of teachers was real. Further, we checked with participants whether the differences in ratings for each question was caused by lying.

Lie-truth group:

(1) To explore the actual mental processes when evaluating the teachers, *we described the purpose of the study as exploring the relationship between eye-tracking indicators and questionnaires surveys. We emphasized that, in addition to the scientific study, our main purpose was to assist the school to investigate the students’ satisfaction with teachers.* We asked participants for their names and student numbers to increase authenticity.
(2) An interview was conducted to ask each participant to describe a teacher they disliked the most during college life. We emphasized that the interview was anonymous and not recorded.
(3) We asked participants to fill out the questionnaire in front of the eye tracker. The teachers that participants mentioned in the interview were evaluated in the questionnaire. The eye tracker recorded all eye behaviors during the questionnaire filling process. *We emphasized that the questionnaire was real-name and would be recorded. Moreover, the teacher they evaluated could see the responses and respondents.*
(4) *After completing the questionnaire, we explained to participants that the scenario of evaluating teachers was simulated.* We told participants that this study aimed to explore the relationship between eye-tracking indicators and deception in questionnaire surveys. The responses of the questionnaire would be completely confidential and anonymous.
(5) *We asked participants to fill out the questionnaire again for the same teachers according to the actual situation.* An irrelevant questionnaire would be interspersed between the two responses to eliminate learning effects.
(6) We confirmed with participants whether they believed the scenario of real-name evaluation of teachers was real. Further, we checked with participants whether the differences in ratings for each question was caused by lying.

**FIGURE 3:**
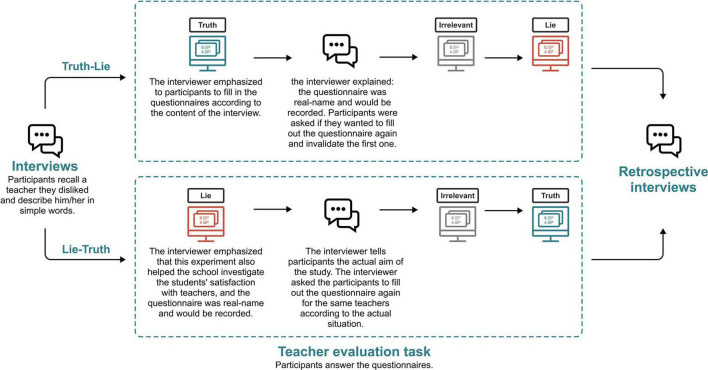
The experiment procedure of Study 2.

The procedure of the experiment is shown in Figure 3.

#### Data Analysis

In Study 2, paired-samples *t*-test was conducted for ratings and pupil size, Wilcoxon signed ranks test was conducted for blinks, saccades, and fixations. In addition to the same data collection and analysis as in Study 1, this study built a lie/truth classifier based on the eye-tracking data obtained in Study 2 to explore the accuracy of eye behaviors in detecting spontaneous lying.

We tried a range of classification algorithms, such as decision tree, discriminant analysis, support vector machine (SVM), nearest neighbor classifier, ensemble classifier, etc. The classifiers were developed using the classification learner in MATLAB. The most promising classification accuracy came from a linear SVM, and the performance of all classifiers is shown in Supplementary Table S1. SVM classifier is a type of supervised machine learning approach that attempts to distinguish between two classes of data points separated by a hyperplane in a high dimensional space (Cortes and Vapnik, 1995; Chen et al., 2020). SVM is widely used to deal with classification problems in machine learning (Byvatov et al., 2003; Peltier et al., 2009), many studies of lie detection (Mottelson et al., 2018; Mazza et al., 2020; Mathur and Matarić, 2020) or eye movements (Huang et al., 2015; Dalrymple et al., 2019; Steil et al., 2019; Kang et al., 2020) have used SVM for classification.

We used cross-validation techniques in which the train and test sets are rotated over the entire data set. We conducted five-fold cross-validation. One fold was used to validate the modal trained using the remaining folds. This process is repeated five times such that each fold is used exactly once for validation. After the preprocessing procedure for data quality control, there were 184 lying trials and 175 truth trials left. The number of lying trials in five folds is 37, 37, 37, 37, and 36, respectively. And the number of truth trials in each fold is 35. To measure how well the predictors work, this study adopted the following evaluation metrics: accuracy, precision, recall, F_1_-score, and area under the curve of receiver operating characteristics (AUC ROC). AUC provides an aggregate measure of performance across all possible classification thresholds. The accuracy, precision and recall relate to true positives (TP), false positives (FP) and false negatives (FN) as:


$$accuracy=\frac{TP+TN}{TP+TN+FP+FN},precision=\frac{TP}{TP+FP},$$

$$recall=\frac{TP}{TP+FN}$$

The F_1_-score can be interpreted as a weighted average of the precision and recall, and is defined as:


$$F_{1}-score=2\times \frac{precision\times recall}{precision+recall}$$

### Results

#### Ratings

94.3% of the participants (33 of 35) believed the scenario of real-name evaluation of teachers was real and chose to lie. Participants gave higher ratings in the real-name condition (*M* = 3.937, SD = 0.820) than that in the honest condition (*M* = 2.790, SD = 1.042). The results of the paired-samples *t*-test showed a significant difference between the ratings for the real-name condition and the honest condition (*t* = 19.934, Cohen’s *d* = 1.070, *p* < 0.001). 68.86% of all questions (241 of 350) were rated higher in the real-name condition, and were confirmed that lies existed by participants. In the spontaneous lie condition, most participants lied slightly to completely.

#### Pupil Size

There was a significant difference in pupil size between deceptive and truthful responses (Cohen’s *d* = 0.858, *p* < 0.001). Lies resulted in larger pupil diameter, as shown in Table 4.

**TABLE 4:** The analysis of pupil size in Study 2.

Variable	Spontaneous lie		Truth		t	Effect size Cohen’s *d*	*P*-value (2-tailed)
	*M*	*SD*	*M*	*SD*			
Pupil diameter (mm)	4.431	0.506	4.347	0.536	3.361	0.858	<0.001***

Significance codes: ***p < 0.001.

#### Blinks

In Study 2, as shown in Table 5, there were no significant differences in count, frequency, average duration, and total duration of blink between deceptive and truthful responses (*p* > 0.05).

**TABLE 5:** The analysis of blink behaviors, saccade behaviors and fixation behaviors in Study 2.

Variable	Spontaneous lie		Truth		*Z*	Effect size *r*	*P*-value (2-tailed)
	*M*	*SD*	*M*	*SD*			
Blink count	1.833	3.698	2.333	3.848	−1.463	−0.106	0.143
Blink frequency (count/s)	0.453	0.865	0.632	1.214	−1.553	−0.112	0.120
Blink duration average (ms)	152.353	221.158	105.125	65.682	−1.933	−0.140	0.053
Blink duration total (ms)	474.554	1309.936	470.167	762.827	−1.523	−0.110	0.128
Saccade count	21.094	12.658	26.740	16.780	−4.151	−0.300	<0.001***
Saccade frequency (count/s)	6.095	2.820	6.646	2.842	−2.716	−0.196	0.007**
Saccade duration average (ms)	46.296	12.501	47.260	9.941	−0.398	−0.029	0.690
Saccade duration total (ms)	994.484	589.597	1280.223	844.124	−4.133	−0.298	<0.001***
Saccade velocity (°/s)	91.999	23.320	91.260	28.526	−0.727	−0.054	0.467
Saccade amplitude (°)	5.048	1.612	5.063	2.176	−0.592	−0.044	0.554
Fixation count	11.040	5.466	11.347	6.629	−0.037	−0.003	0.971
Fixation frequency (count/s)	3.273	1.132	3.038	1.268	−2.265	−0.171	0.024*
Fixation duration average (ms)	155.482	48.075	136.936	41.516	−4.531	−0.342	<0.001***
Fixation duration total (ms)	1708.461	934.102	1604.671	1104.755	−1.902	−0.143	0.150

Significance codes: ***p < 0.001, **p < 0.01, *p < 0.05.

#### Saccades

There were no differences in average duration, velocity, and amplitude of saccade (*p* > 0.05). The differences in saccade count (*r* = −0.300, *p* < 0.001), saccade frequency (*r* = −0.196, *p* = 0.007) and total saccade duration (*r* = −0.298, *p* < 0.001) between deceptive and truthful responses were statistically significant. When lying, the saccade count, the saccade frequency, and the total saccade duration were significantly lower (see Table 5).

#### Fixations

As can be seen from the Table 5, there were no significant differences in fixation count, total fixation duration between deceptive and truthful responses (*p* > 0.05), while there were significant differences in fixation frequency (*r* = −0.171, *p* = 0.016) and average fixation duration (*r* = −0.342, *p* < 0.001). Deception caused increased fixation frequency and average fixation duration.

The fixation behaviors in AOIs were analyzed (see Table 6). In the QT area, there were no significant differences in fixation count between deceptive and truthful responses (*p* > 0.05). However, when lying, the percentage fixation time (*r* = −0.233, *p* = 0.002) was significantly higher. In the NO area, the fixation count (*r* = −0.340, *p* < 0.001) and the percentage fixation time (*r* = −0.354, *p* < 0.001) were significantly lower when lying. The fixation count was significantly lower in the NK area when lying (*r* = −0.152, *p* = 0.043), while the percentage fixation time was no significant differences (*p* > 0.05). In the PO and PK areas, when in the lying condition, the fixation count (*r*_*PO*_ = −0.428, *r*_*PK*_ = −0.323, *p* < 0.001) and the percentage fixation time (*r*_*PO*_ = −0.487, *r*_*PK*_ = −0.458, *p* < 0.001) were significantly higher than when in honesty condition. In the EO area, lies caused increased fixation count (*r* = −0.195, *p* = 0.010) and percentage fixation time (*r* = 0.199, *p* = 0.008). Meanwhile, in the MO area, lies resulted in higher fixation count (*r* = −0.149, *p* = 0.048).

**TABLE 6:** The analysis of fixation behaviors in the AOIs in Study 2.

AOI	Variable	Spontaneous lie		Truth		*Z*	Effect size *r*	*P*-value (2-tailed)
		*M*	*SD*	*M*	*SD*			
QT	Fixation count	5.648	3.592	6.097	4.563	−0.663	−0.050	0.508
	The percentage fixation time (%)	25.501	15.641	21.488	15.736	−3.085	−0.233	0.002**
NO	Fixation count	0.080	0.292	0.455	1.089	−4.509	−0.340	< 0.001***
	The percentage fixation time (%)	0.246	1.0211	1.949	5.198	−4.695	−0.354	< 0.001***
NK	Fixation count	0.364	0.774	0.523	0.913	−2.019	−0.152	0.043*
	The percentage fixation time (%)	1.328	2.666	1.810	3.485	−1.195	−0.090	0.232
PO	Fixation count	0.858	1.194	0.267	0.661	−5.684	−0.428	< 0.001***
	The percentage fixation time (%)	4.380	6.875	0.895	2.580	−6.466	−0.487	< 0.001***
PK	Fixation count	0.881	0.958	0.494	1.025	−4.282	−0.323	< 0.001***
	The percentage fixation time (%)	4.194	5.330	1.525	3.168	−6.074	−0.458	< 0.001***
EO	Fixation count	0.972	1.239	0.699	1.217	−2.588	−0.195	0.010*
	The percentage fixation time (%)	4.676	7.061	3.103	6.188	−2.636	−0.199	0.008**
MO	Fixation count	2.954	2.866	3.500	3.046	−1.975	−0.149	0.048*
	The percentage fixation time (%)	14.587	12.393	13.085	11.482	−0.170	−0.013	0.242

*AOI, area of interest; QT, the question text; NO, the extreme negative option; NK, the negative keyword; PO, the extreme positive keyword, PK, the positive keyword; EO, the extreme options; MO, the medium options.*

Significance codes: ***p < 0.001, **p < 0.01, *p < 0.05.

**FIGURE 4:**
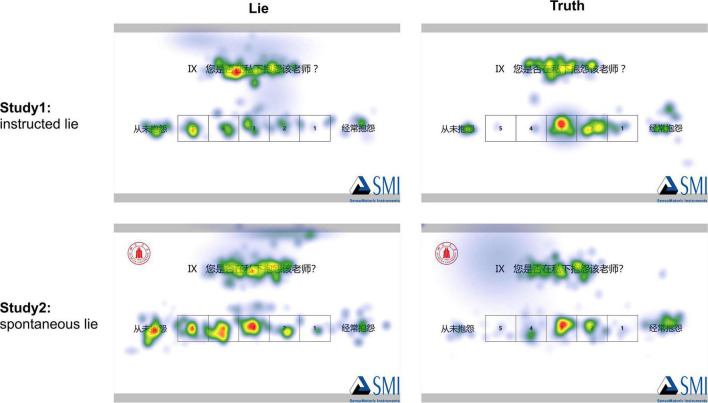
The heat map of lying and honesty in Study 1 and Study 2.

The heat map of lie and truth in Study 1 and Study 2 is shown in Figure 4.

#### Classification

We built a linear SVM, the kernel function is linear, the kernel scale is 1, the box constraint level is 2, and the multiclass method is one-vs-one.

Features were chosen from a previous study in classifying deception in personality tests (van Hooft and Born, 2012), such as fixation behaviors in AOIs. In Study 1 and Study 2, fixation behaviors and pupil size showed significant differences and consistent tendencies. Hence, this study also included them based on the results of Study 1 and Study 2. The feature groups pupil size, fixation behaviors, and fixation behaviors in AOIs presented the most viable features for classifying truths and lies, and were thus shown in our final classifier (see Table 7).

**TABLE 7:** Feature groups and specific features for each group.

Feature group	Features
Pupil size	Pupil diameter
Fixation behaviors	Fixation count, fixation frequency, fixation duration total, fixation duration average
Fixation behaviors in AOIs	Fixation count, fixation time percentage in the QT, NO, NK, PO, PK, EO, and MO areas

*AOIs, areas of interest; QT, the question text; NO, the extreme negative option; NK, the negative keyword; PO, the extreme positive keyword, PK, the positive keyword; EO, the extreme options; MO, the medium options.*

As reported in Table 8, using randomized five-fold cross-validation, this study obtained an accuracy of 74.09%. The rates of precision are 0.77 and 0.72 for lie and truth, respectively. The rates of recall are 0.71 and 0.78 for lie and truth, respectively. These produced an average F1-score of 0.74 for lie and 0.75 for truth. The ROC AUC of both lie and truth is 0.78. The scores showed the mean score from cross-validation.

**TABLE 8:** Confusion matrix and performance metrics of classification results.

Accuracy (%)	74.09%	True label		Precision	F_1_-score	ROC AUC
		Lie	Truth			
Predicted label	Lie	130	39	0.77	0.74	0.78
	Truth	54	136	0.72	0.75	0.78
Recall		0.71	0.78			

*ROC AUC, area under the curve of receiver operating characteristics.*

### Discussion

In Study 2, when lying, the participants had larger pupil size, greater fixation count, and duration, which supported the theory that cognitive load is higher in the dishonest condition. However, when the participants were lying, they showed lower count, velocity, and amplitude of saccade, contradicting the theory that deception increased cognitive load. There were no significant differences in blink behaviors in Study 2. The results of saccade and blink behaviors were inconsistent with Study 1.

According to the results based on AOIs analysis. When lying, participants paid less attention to the NO and NK areas, while more to the PO and PK areas. In general, it is consistent with mental processes of wanting to rate higher under the condition of real-name evaluation. Moreover, in the QT area, the percentage fixation time was higher when lying, indicating that cognitive load was higher when lying. What is more, participants focused more on the EO area when lying, and more on the MO area when honesty, which is in line with the study from van Hooft and Born (2012).

The linear SVM classifier performed well over both chance level and human performance. The accuracy this study achieved showed that eye-tracking data could be a promising path toward deception detection.

## General Discussion

In two studies, we have investigated whether we can assess deception in questionnaire surveys with eye-tracking. Here, to explore if the differences of eye behaviors between lying and honesty were consistent and generalizable to reality, this research compared the results of Study 1 (instructed lying) and Study 2 (spontaneous lying).

The significance and tendency of data on pupil size and fixation across the two studies were all consistent. When lying, the participants had increased pupil size, fixation count, fixation duration. The pupil and fixation data in this study support the opinion that deception is more cognitively demanding. The elevated mental effort in lying can elicit a higher-than-normal level of nervousness and anxiety (Gonzales, 2018), and elevated mental effort leads to pupil dilation and more fixation behaviors. Consistent with previous studies, higher cognitive load caused by deception resulted in more fixation behaviors (Chen et al., 2011; Zagermann et al., 2016), and generated larger pupil size (Wang et al., 2010; Cook et al., 2012; Proudfoot et al., 2015, 2016). Meanwhile, the larger pupil size when lying may also be caused by memory retrieval (Otero et al., 2011; Kucewicz et al., 2018), increased arousal (Bradshaw, 1967), etc.

According to the analysis of AOIs, both Study 1 and Study 2 showed that the participants focused more on positive items when lying, and focused more on the negative items when being honest. The analysis of the ratings revealed that participants gave higher ratings when lying, consequently, it is logical to focus more on positive items. Surprisingly, in Study 2, there were also significant differences in the QT, EO, and MO areas, but not in Study 1. Deception is a cognitively demanding task, which is reflected in increased percentage fixation time in the QT area when lying in Study 2, the participants needed more effort to read question text. Meanwhile, the participants paid more attention to the EO area and paid less attention to the MO area when lying. This may be since in Study 2, when participants spontaneously lied, the motivation to lie was stronger because of morality and fear of negative consequences if the teacher found out. In this condition, the participants may have made decisions directly based on social desirability without memory recall, resulting in a greater focus on the extreme options when lying, consistent with the previous study (van Hooft and Born, 2012).

The blink behaviors only showed significance in Study 1. The count, frequency, and total duration of blink were significantly greater when lying in Study 1. Previous studies showed that blink count correlated with deceit George et al. (2017), Borza et al. (2018) found that dishonest caused increased blink count and duration. However, increasing blink count also means decreasing cognitive load, arousal level, attentional load (Zagermann et al., 2016; Maffei and Angrilli, 2018). The higher cognitive load in Study 2 may lead to decreased blink behaviors. The combined effect of these factors has contributed to the non-significance in blink behaviors of Study 2. The blink behaviors in deception conditions are complex and still need further research.

There were significant differences in saccade behaviors in both Study 1 and Study 2, whereas the tendency was the opposite. When lying, the saccade count, saccade velocity, and saccade amplitude were higher in Study 1 and were lower in Study 2. Vrij et al. (2015) found that saccade velocity was higher when lying. But in this research, the results of Study 1 and Study 2 are different. The differences may indicate that saccade is not a reliable predictor of deception in questionnaire surveys, which has also been mentioned in a previous Study by Borza et al. (2018).

Based on the comparative analysis of Study 1 and Study 2, it can be concluded that the pupil size and fixation behaviors are more reliable and valid for lie detection in the actual situation. The pupil size and fixations are not restricted to explicitly instructed lying, but can also be observed for spontaneous lying. However, the blink and saccade behaviors showed different or even contradictory performances in Study 1 and Study 2, which still need further research. Results from two studies showed that the mental processes of instructed lying are likely not the same as the actual spontaneous one. These differences may be caused due to the more complex mental processes of deception in the actual situation. The findings expanded our understanding of the detection of the spontaneous lie. This study has identified eye movement predictors that remain valid in spontaneous lying.

The current study built a linear SVM classifier on eye-tracking data in Study 2. This study achieved an F_1_-score of 0.74 for lie detection, and an F_1_-score of 0.75 for truth detection. The eye behaviors can help to detect lie with an accuracy of 74.09%. The results of the experiment showed that eye behaviors are good predictors of deception in questionnaire research. Although van Hooft and Born (2012) achieved 82.9% accuracy in lie detection in the personality test questionnaire, the participants in the personality test were instructed to lie. In the actual situation, the mental processes of spontaneous deception are more complex, the indicators of eye behavior can be influenced by other emotions, such as arousal, fear, guilty. This may lead to the decline of accuracy. Despite this, the classifier in this study outperformed both chance level and human performance by a wide range. In addition to the previously verified lie detection in personality test questionnaire surveys, eye behaviors are also valid for detecting lies in questionnaire surveys that evaluate others. It showed the potential of eye-tracking technology for detecting lies in questionnaire surveys.

The present study contributes to understanding the relationship between deception and eye behaviors and provides a basis for detecting lies in questionnaire surveys. Eye-tracking can help improve the quality of the questionnaire. Before publishing the questionnaire, the publishers can test it with a small sample by asking respondents to fill it in on the eye-tracker. According to the eye-tracking data, the high-sensitivity questions can be found, and the reliability of the answers obtained for each question can be evaluated. There are some ways to reduce the incorrect results due to deliberate misreporting, such as changing the question wording and frame, increasing the respondent’s privacy, etc. (Tourangeau and Yan, 2007; Krumpal, 2013). Using these methods, questionnaire publishers can modify the high-sensitivity questions or the survey mode, preventing respondents from lying because of social desirability factors. When referring to questionnaire survey results, eye-tracking can also be used to evaluate the reliability of the questionnaire to avoid using unreliable survey results.

The current study also has limitations that motivate future investigations. Despite we confirmed with participants several times to ensure that they had given honest answers in the honesty condition, and removed the data that they felt dishonest. However, participants still possibly have modified their answers to some extent due to social desirability or reluctance to admit lying, so this study still cannot guarantee complete honesty of the answers. Although the accuracy of lie detection by the classifier is much higher than human performance, it is still unable to support reliable binary lie classification. Moreover, whether the eye behavior indicators are still valid for lie detection in less sensitive questionnaires needs further study. Future studies can focus on more behavioral and implicit parameters to enhance lie detection accuracy in questionnaire surveys, such as electroencephalogram, face-reading, and mouse-tracking.

## Conclusion

This study explored the feasibility of eye-tracking for lie detection in questionnaire surveys. The eye behaviors in instructed lying and spontaneous lying conditions were investigated separately. Compared to previous studies on lie detection in questionnaire surveys, this study incorporated spontaneous lies in the actual situation. Because the participants experienced more natural and complete mental processes, the eye-tracking data were more reliable.

Through the two studies, the following conclusions were drawn: Eye-tracking signatures of lying are not restricted to instructed deception, but are also applicable to spontaneous deception. Pupil size and fixation behaviors were found to be useful in identifying lies in questionnaire surveys, while blink and saccade behaviors were not. When lying, respondents have larger pupil size, higher fixation count and duration. The results also showed that respondents paid attention to different areas of the questionnaire when lying and when they were honest. Furthermore, the deception classifier based on eye behaviors obtained convincing classification rates (74.09%) of lies in the actual lie situation. Those findings can provide anticipatory help to questionnaire publishers.

## Data Availability Statement

The raw data supporting the conclusions of this article will be made available by the authors, without undue reservation.

## Ethics Statement

The studies involving human participants were reviewed and approved by Sichuan University. The patients/participants provided their written informed consent to participate in this study.

## Author Contributions

XF contributed to the conceptualization, investigation, methodology, formal analysis, wrote the manuscript, and revised the manuscript. YS contributed to the conceptualization, investigation, methodology, formal analysis, and revised the manuscript. XZ, XW, and XD contributed to the investigation and revised the manuscript. MW contributed to the conceptualization, investigation, methodology, resources, supervision, and revised the manuscript. All authors contributed to the article and approved the submitted version.

## Conflict of Interest

The authors declare that the research was conducted in the absence of any commercial or financial relationships that could be construed as a potential conflict of interest.

## Publisher’s Note

All claims expressed in this article are solely those of the authors and do not necessarily represent those of their affiliated organizations, or those of the publisher, the editors and the reviewers. Any product that may be evaluated in this article, or claim that may be made by its manufacturer, is not guaranteed or endorsed by the publisher.
