# Supplementary material for: Assessing Deception in Questionnaire Surveys With Eye-Tracking
Source: Front Psychol. 2021 Nov 22;12:774961. doi: 10.3389/fpsyg.2021.774961 (PMC8646095; doi:10.3389/fpsyg.2021.774961)
Supplement: Supplementary file 1 [file Table_1.pdf]

Supplementary Material

Table S1 Performance metrics of decision trees, discriminant analysis, support vector machine (SVM), nearest neighbor classifier, ensemble classifier and logistic regression classifier. The performance obtained by each classifier in the 5-flod cross-validation.

Classifier		Accuracy	Precision	Recall	F1-score	ROC AUC
Decision trees	Lie	65.18%	0.73	0.66	0.69	0.72
	Truth		0.67	0.74	0.71	0.72
Discriminant analysis	Lie	69.92%	0.73	0.66	0.69	0.77
	Truth		0.67	0.74	0.71	0.77
SVM	Lie	74.09%	0.77	0.71	0.74	0.78
	Truth		0.72	0.78	0.75	0.78
Nearest neighbor classifier	Lie	69.92%	0.71	0.71	0.71	0.74
	Truth		0.69	0.69	0.69	0.74
Ensemble classifier	Lie	72.70%	0.75	0.70	0.72	0.77
	Truth		0.70	0.76	0.73	0.77
Logistic regression classifier	Lie	72.14%	0.74	0.70	0.72	0.78
	Truth		0.70	0.74	0.72	0.78

ROC AUC, area under the curve of receiver operating characteristics.
